# Behaviour problems of patients with bipolar disorder

**DOI:** 10.1192/j.eurpsy.2021.525

**Published:** 2021-08-13

**Authors:** I. Kammoun, N. Halouani

**Affiliations:** Psychiatry B, Hedi Chaker UHC, Sfax, Tunisia

**Keywords:** bipolar disorder, behaviour problems, Risk factors, psychoactive substance use

## Abstract

**Introduction:**

Bipolar disorder is associated, in popular belief, with violence as any psychiatric pathology. Studies in recent years have found an increased risk of violence in patients with bipolar disorder.

**Objectives:**

To describe the socio-demographic characteristics of bipolar patients and identify the various risk factors for violence.

**Methods:**

We conducted a retrospective descriptive, analytical study, including patients with bipolar disorder type I and II in the CHU HEDI CHAKER Sfax psychiatry department whose reason for hospitalization was hetero-aggressiveness during a period of 6 months ranging from 1 January to 30 June 2019.

**Results:**

We’ve collected 32 patients. The average age of our sample is 36 years. Half of the patients (50%) were single. Most of these patients were unemployed. The type of bipolar disorder was dominated by type I (90.3%) in a manic episode. These patients had antisocial pathological personality in 18.8% and borderline personality in 20%. Siblings and ascendants accounted for 68.7% of victims of violence. Our study showed that comorbidity to the use of psychoactive substances was present in 65.6%. The exaltation of mood was intense in 78.1% with a bad insight in 75%. Patients with violent behaviour were discontinued in 96.9% of cases with poor therapeutic adherence in 90.6% and irregular follow-up in 68.8%. Violence was significantly associated with psychoactive substance use with p=0.037.

**Conclusions:**

The risk of violence in patients with bipolar disorder is higher than in the general population. This risk is particularly high if there was an association with substance abuse and personality disorders.

